# Disparities in paediatric radiology research publications from low- and lower middle-income countries: a time for change

**DOI:** 10.1007/s00247-023-05762-y

**Published:** 2023-09-29

**Authors:** Amaka C. Offiah, Omolola M. Atalabi, Monica Epelman, Geetika Khanna

**Affiliations:** 1 Division of Clinical Medicine, University of Sheffield, Sheffield Children’s NHS Foundation Trust, Room 3, Damer Street Building, Western Bank, Sheffield, S10 2TH UK; 2 Department of Radiology, Sheffield Children’s NHS Foundation Trust, Sheffield, UK; 3 Department of Radiology, University College Hospital, Ibadan, Oyo State Nigeria; 4 Department of Radiology, Nicklaus Children’s Hospital, Miami, FL USA; 5 Department of Radiology & Imaging Sciences, Emory University and Children’s Healthcare of Atlanta, Atlanta, GA USA

**Keywords:** Diversity, Equity, Health research, Inclusion, Research pathway

## Abstract

The positive impact of diversity on health research and outcomes is well-recognised and widely published. Despite this, published evidence shows that at every step of the research pathway, issues of equity, diversity and inclusion (EDI) arise. There is evidence of a lack of diversity within research teams, in the research questions asked/research participants recruited, on grant review/funding panels, amongst funded researchers and on the editorial boards and reviewer pools of the journals to which results are submitted for peer-reviewed publication. Considering the journal *Pediatric* *Radiology*, while its editorial board of 92 members has at least one member affiliated to a country in every region of the world, the majority are in North America (*n*=52, 57%) and Europe (*n*=30, 33%) and only two (2%) are affiliated to institutions in a lower middle-income country (LMIC) (India, Nigeria), with one (1%) affiliated to an institution in an upper middle-income country (UMIC) (Peru) and none in a low-income country (LIC). *Pediatric Radiology* is “…the official journal of the European Society of Paediatric Radiology, the Society for Pediatric Radiology, the Asian and Oceanic Society for Pediatric Radiology and the Latin American Society of Pediatric Radiology”. However, of the total number of manuscripts submitted for potential publication in the four years 2019 through 2022, only 0.03% were from a LIC and only 7.9% were from a LMIC. Further, the frequency of acceptance of manuscripts from UMIC was seven times higher than that from LMIC (no manuscripts were published from LIC). Increased collaboration is required between researchers across the globe to better understand the barriers to equity in the funding, conduct and publication of research from LIC and LMIC and to identify ways in which we can overcome them together.

**Graphical abstract:**

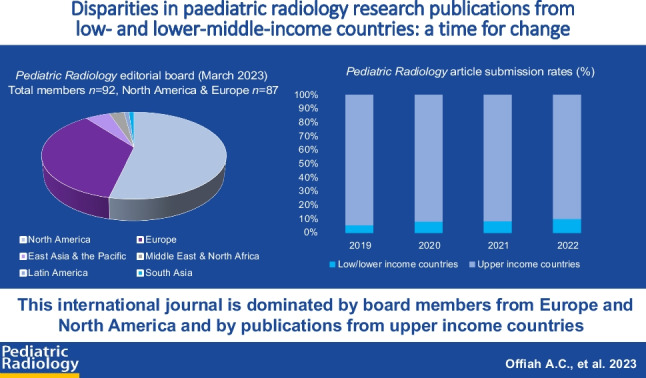

## Introduction

We live in a time of increasing awareness of the disparities in research funding and research outcomes between individuals, societies and countries. Some disparities are related to lack of resources impacting quality of equipment, training and experience, while others are related to systemic biases in academia [1, 2].

The impact of diversity (or the lack thereof) on health research is well-recognised and widely documented. For example, the National Institute on Minority Health and Health Disparities (a section of the National Institute of Health created in 2000 to “lead scientific research to improve minority health and eliminate health disparities”) discusses and provides some real-world examples of the importance of diversity and inclusion in clinical trials [3].

**Fig. 1:**
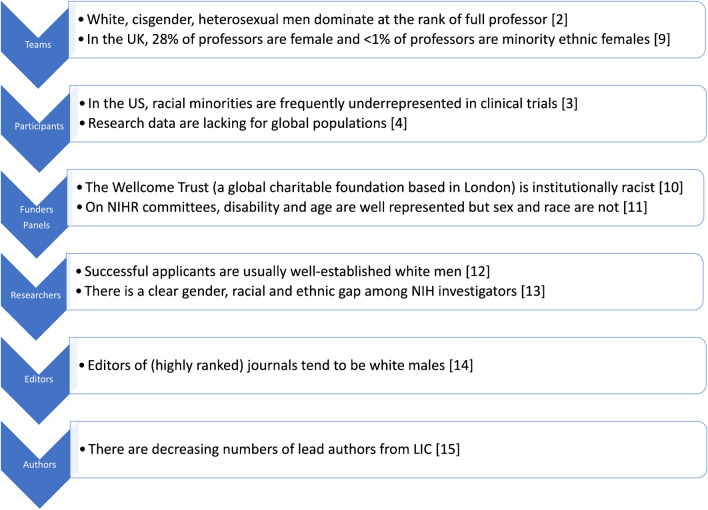
Lack of diversity in the research pathway. In every step of the research pathway, there is evidence of a lack of sex, gender and racial diversity [8–15]. *LIC* low-income economy countries, *NIH* National Institutes of Health, *NIHR* National Institute for Health and Care Research, *UK* United Kingdom, *US* United States

At every step of the research pathway, issues of equity, diversity and inclusion (EDI) arise. There is evidence of a lack of diversity within research teams, particularly at senior levels [4], in the research questions asked/research participants recruited [5], on grant review/funding panels [6], amongst funded researchers [7] and on the editorial boards and reviewer pools of the journals to which results are submitted for peer-reviewed publication (Fig. 1) [8, 10]. These issues must be addressed but there should be no concern that EDI efforts will come at the cost of compromising quality in research and scientific writing. Instead, emphasis should be on putting measures in place to achieve equity, removing subjectivity when possible from the various steps of the research pathway and on training those who are traditionally underrepresented, providing them with the resources needed to achieve equity.

While diversity issues can be related to sex, gender, race/ethnicity, socioeconomic status, academic affiliation and other factors, the remainder of this article focuses on diversity as it relates to low-income countries (LIC) and lower middle-income countries (LMIC). The World Bank classifies all countries of the world into 4 groups—LIC, LMIC, upper middle-income (UMIC) and high-income economy (HIC) countries—based on their gross national income per capita:  <$1,135, $1,136–$4,465, $4,466–$13,845 and  >$13,846, respectively [16]. For specific initiatives which the editors of *Pediatric Radiology* suggest might address some of these issues, the reader is referred to the editorial by Offiah et al. [17].

## Contributions to international journals from low/low-middle-income countries

### High-impact non-radiology medical journals

In this section, we summarise evidence from five studies that evaluate authors’ country affiliations—first, articles published in high-impact journals (presumably the aspiration of most researchers); second, articles where the research was based in LIC/LMIC (being more likely to have authors from LIC/LMIC): third, articles related to global health (being relevant to all countries irrespective of income); fourth, articles related to infectious diseases (being more prevalent in LIC/LMIC); and fifth, articles related to paediatrics (being of some relevance to the readers of this journal).

Merriman et al. [18] reviewed the first 20 research and the first 20 non-research articles published from January 2018–June 2019 in 14 high-impact journals (general medical journals *n*=6, specialist global health journals *n*=8). They found that first author/last author affiliations with institutions in HIC, MIC and LIC/LMIC accounted for 69%/74%, 19%/16% and 5%/5%, respectively, for research articles and 82%/82%, 13%/14% and 2%/2%, respectively, for non-research articles [18]. The total number of first and last authors from Asia, Latin America, the Caribbean, the Middle East, North Africa, Oceania and sub-Saharan Africa (*n*=242) was lower than the total number of first and last authors from either Europe (*n*=300) or North America (*n*=403) alone.

In a recent article authored by Shambe et al. (who reviewed 882 articles published between January 2016 and December 2020 with a total of 10,570 authors), publications in high-impact journals and those involving multiple countries were less likely to have authors affiliated to institutions in LMIC [19].

In a large review of the diversity in authorship of 786,779 articles related to global health published between 2000 and 2017, Dimitris et al. [20] found that 86% had at least one LMIC author and that the percentage with a first/last author from a LMIC was 77% and 71%, respectively. However, when the publications were about LMIC, these findings reduced to 59%, 37% and 29%, respectively. Furthermore, the increase over time of publications that included an author with a LMIC affiliation was accounted for by an increase in publications/representation from authors affiliated to UMIC and not by those affiliated to LIC [21].

In their systematic review, Modlin et al. [22] found that despite an increase in the amount of infectious disease research conducted in LIC between 1998 and 2018, author metrics showed a decreasing trend in first or last authorship affiliated with institutions in LIC. The highest and lowest proportions of first/last authors from LIC were from the Asian-Pacific region and Eastern Europe, respectively. A low proportion of first/last authors from Latin America was also recorded. Approximately 50% of first/last authors from LIC had dual affiliation, with the second affiliation almost universally being with a HIC—the top three first/last author percentages were for affiliations with the United States (US), United Kingdom (UK) and France, being 55%/52%, 13%/15% and 6%/7%, respectively. Modlin et al. also showed that of research studies that were conducted in Africa, first and last authors were from Africa in only 50% and 41%, respectively [22].

Concerning authorship on papers related to paediatric research, Rees et al. [21] reviewed 1,243 articles which met their inclusion criteria from a total of 24,169 articles published between 2006 and 2015. They found “authorship parasitism” (articles having no authors from the study country) to be rare. However, most of the 9,876 authors were from UMIC (42%) and HIC (33%) compared to 16% and 5% from LMIC and LIC, respectively. Furthermore, when they considered those articles from LIC, a significant proportion of first and last authors were affiliated to institutions in HIC compared to those affiliated to institutions in LIC [21].

### Radiology journals

While there are publications reporting on the relative lack of sex and racial diversity amongst radiologists, underrepresentation of women in editorial boards of radiology journals, reduced access to radiology services by ethnic minorities and means by which these various disparities might be improved [23–32], there are very few radiology articles assessing diversity as it relates to the economic status of the countries in which radiology research is either performed or where the authors are based. In 2006, Miguel-Dasit et al. showed that for oral abstracts presented at the 2000 European Congress of Radiology, the probability of subsequent full publication was significantly impacted by the first authors’ country of affiliation [33]. There were no abstracts/publications from LIC or LMIC.

Journal editors are responsible for the final content and quality of the journal’s articles, while editorial boards consist of field experts who advise and support the editor(s) in several ways including identifying new topics and reviewing submitted manuscripts [34]. The diversity of a journal’s editors and editorial board is therefore likely to impact the diversity of that journal’s publications. We found very limited literature specifically looking at authorship or editorial board membership of radiology journals in relation to income of the countries to which they are affiliated. Therefore, we visited the websites of five radiology journals (including *Pediatric* *Radiology*) to review the makeup of their editorial boards as of March 2023.

Based on the Scimago Journal and Country Ranking, the top three radiology journals in 2021 were *JACC Cardiovascular Imaging*, *Radiology: Artificial Intelligence* and *Radiology* [35]. According to its website, *JACC Cardiovascular Imaging* has 86 editors and editorial board members from 13 countries/regions. Of these editors and editorial board members, 66 (77%) are affiliated to institutions in North America, 15 (17.4%) to institutions in Europe, two (2.4%) to institutions in the Middle East (Saudi Arabia and United Arab Emirates) and one each (1.2%) to institutions in East Asia and the Pacific (Australia), South Asia (India) and Latin America (Mexico). Of the 86 editors and editorial board members, one is affiliated to an UMIC (Mexico) and one to an LMIC (India). None is affiliated to a LIC [36].

**Fig. 2:**
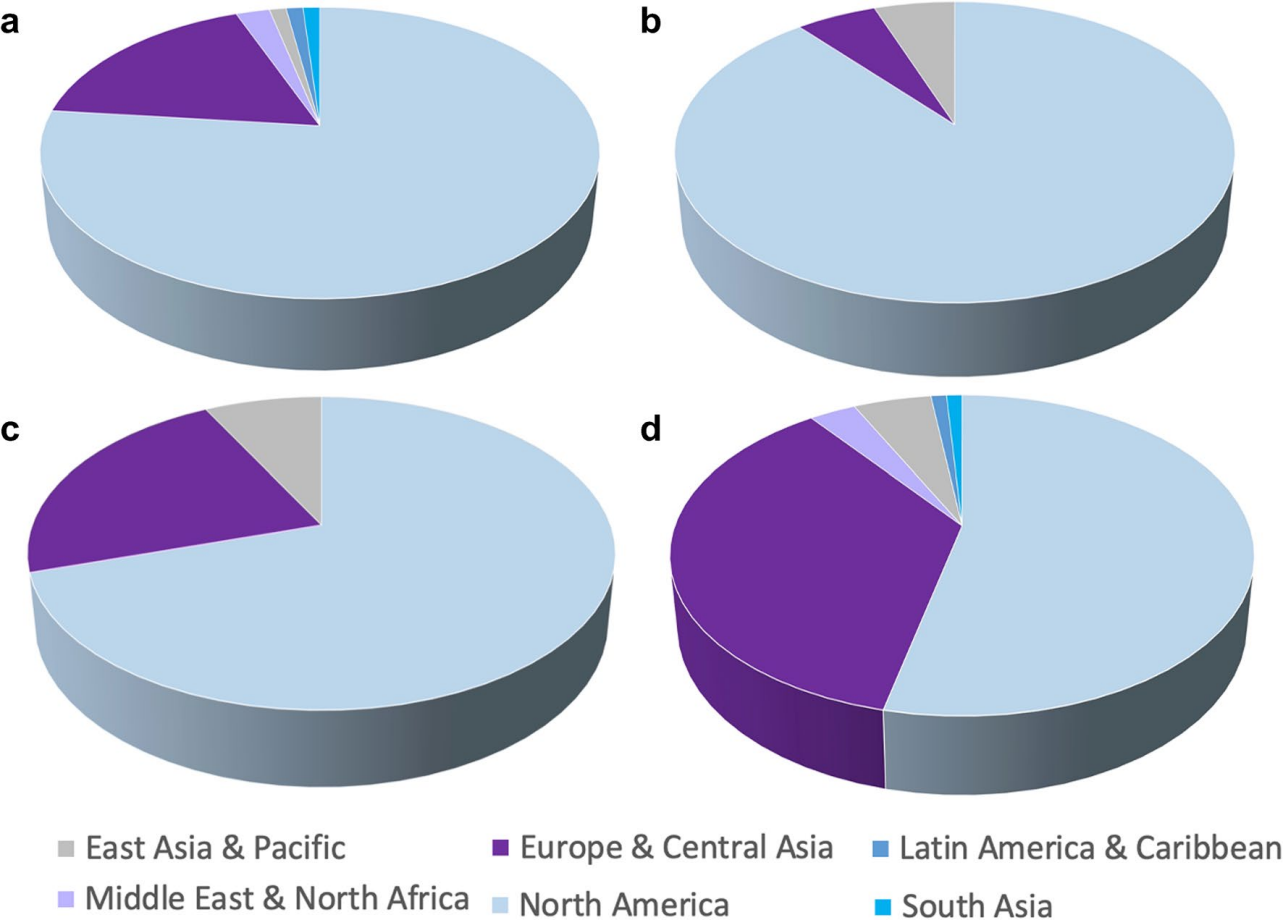
Affiliations of editorial board members of the top three radiology journals (according to Scimago Journal and Country Ranking [35]) (**a**–**c**) and of “*Pediatric* *Radiology*” [39] (**d**) as obtained from their websites on 19th March 2023. **a** “*JACC Cardiovascular Imaging*” [36]. **b** “*Radiology: Artificial Intelligence*” [37]. **c** “*Radiology*” [38]

The editorial board of *Radiology: Artificial Intelligence* has sex and ethnic diversity (based on photographs and names); however of the 36 members, 32 (89%) are affiliated to institutions in North America, 2 (6%) to institutions in Europe and Central Asia (Netherlands, United Kingdom) and 2 (6%) to institutions in Eastern Asia and the Pacific (Japan in both instances). None is affiliated to institutions in UMIC, LMIC or LIC [37]. The distribution of editorial board members of the journal *Radiology* reflects that of the board for *Radiology: Artificial Intelligence*. According to its website on 19th March 2023, of the 105 editorial board members—including four emeritus editors, 73 (70%) are affiliated to institutions in North America, 23 (22%) to institutions in Europe and Central Asia and 8 (8%) to institutions in East Asia and the Pacific (Republic of Korea *n*=5, Japan *n*=3). There are one (1%) and zero affiliated to LMIC (Brazil) and LIC, respectively [38]. Figure 2 illustrates these distributions.

When evaluating these data, it should be borne in mind that these journals are the official journals of American societies (namely, American College of Cardiology and Radiological Society of North America). Hence, it is not unexpected that most of their editorial board members are from North America. So, we also looked at the editorial board composition of the highest ranked international imaging journal on the Scimago Journal and Country Ranking, *Ultrasound in Obstetrics &* *Gynecology*, the official journal of the International Society of Ultrasound in Obstetrics and Gynecology (ISUOG), to find no representation of LIC/LMIC on the editorial board of this international journal [40].

Meshaka et al. evaluated the distribution of abstracts presented at international pediatric radiology conferences, including the annual meetings of the European Society of Pediatric Radiology, the Society for Pediatric Radiology and the International Pediatric Radiology conferences between 2013 and 2016. They noted that 99% of abstracts were from HIC or HMIC and LMIC/LIC accounted for 1% of presented abstracts. The data were similar when evaluating the number of presented abstracts that were converted to published manuscripts by October 2021. There were two publications from LMIC, 19 from UMIC and 412 published manuscripts from HIC [41].

### The journal “*Pediatric Radiology*”

The *Pediatric* *Radiology* journal website states that it is “…the official journal of the European Society of Paediatric Radiology, the Society for Pediatric Radiology, the Asian and Oceanic Society for Pediatric Radiology, and the Latin American Society of Pediatric Radiology” [39]. Its editorial board members’ affiliations would therefore be expected to reflect this diversity. As of 1st April 2023, there were 92 editors [42]. Of these, 52 (57%) were affiliated to institutions in North America and 35 (34%) to institutions in Europe. The editorial board team has at least one member with an affiliation in six of the seven regions of the world—five (5%) in East Asia and the Pacific (Hong Kong *n*=3, Korea Republic *n*=2), three (3%) in the Middle East and North Africa (Israel, Qatar, United Arab Emirates) and one (1%) each in Latin America (Peru) and South Asia (India). Though one of the journal editors is affiliated to an institution in sub-Saharan Africa (Nigeria), there currently are no editorial board members from Africa. We also note that there is no one on our editorial board affiliated to an institution in the Caribbean. *Pediatric* *Radiology* also has no editorial board member affiliated to a LIC (Fig. 2).

**Fig. 3:**
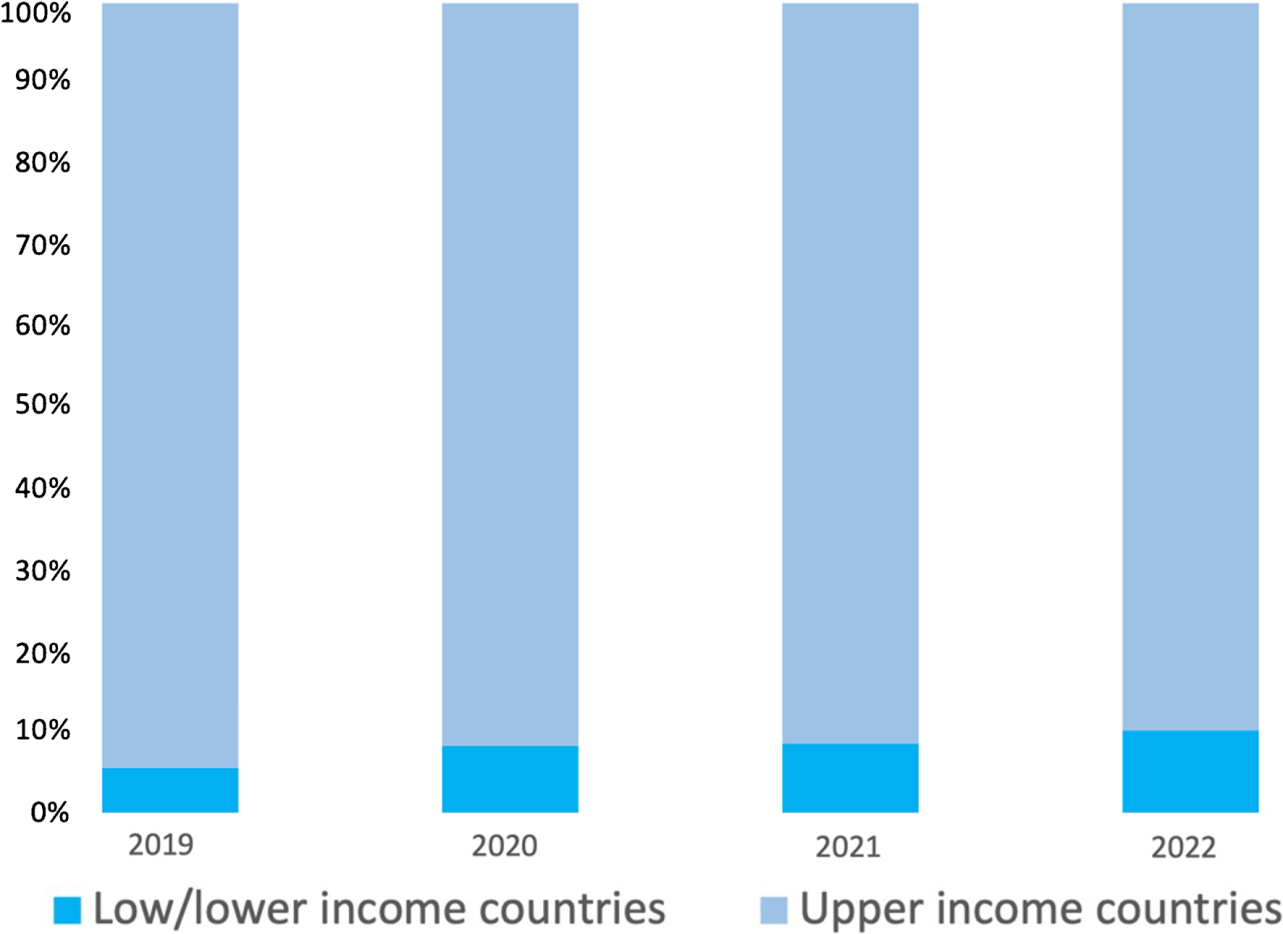
Article submission frequencies (%) to *Pediatric* *Radiology* 2019–2022 subdivided by country income classification

In addition to considering the economic diversity of countries represented by the editorial board of *Pediatric* *Radiology*, we should also consider the diversity of countries represented by submitted and published articles. Although the information is available to the editors, manuscript submission numbers and frequencies of acceptance are business confidential, and Springer (the publisher of *Pediatric* *Radiology*) does not permit their disclosure. However, for the years 2019–2022 inclusive, 0.03% and 8.2% of articles submitted to the journal were from LIC and LMIC respectively (Table 1, Fig. 3), with overall submissions doubling in number over the 4-year period. The total numbers of manuscripts published in *Pediatric* *Radiology* in the same four-year period from LIC/LMIC were zero and 13 respectively.

**Table 1:** Percentage of manuscripts submitted to/published in *Pediatric Radiology* from low- and lower-middle income countries (2019–2022). Only countries that submitted at least one paper over the four years 2019 through 2022 are listed. It is noteworthy that there were no submissions from Bolivia or the few Latin American countries that are classified as lower middle-income economies

Country	2019 (%)		2020 (%)		2021 (%)		2022 (%)	
	Submitted	Published	Submitted	Published	Submitted	Published	Submitted	Published
Bangladesh	0.0	0.0	0.0	0.0	0.1	0.0	0.0	0.0
Bhutan	0.0	0.0	0.0	0.0	0.1	0.0	0.0	0.0
Cameroon	0.3	0.0	0.2	0.0	0.0	0.0	0.0	0.0
Arab Republic of Egypt	1.0	0.0	1.0	0.0	1.0	0.0	1.9	0.8
Ghana	0.0	0.0	0.0	0.0	0.0	0.0	0.1	0.0
India	1.7	0.4	3.9	0.6	4.1	0.3	4.3	0.8
Indonesia	0.0	0.0	0.1	0.0	0.3	0.0	0.0	0.0
Islamic Republic of Iran	1.1	0.0	2.2	0.0	1.7	0.0	2.2	0.0
Kenya	0.0	0.0	0.0	0.0	0.0	0.0	0.1	0.0
Lebanon	0.3	0.0	0.0	0.0	0.1	0.0	0.0	0.0
Morocco	0.1	0.0	0.2	0.0	0.1	0.0	1.9	0.0
Nigeria	0.3	0.4	0.2	0.3	0.3	0.0	0.7	0.8
Pakistan	0.3	0.0	0.2	0.0	0.4	0.0	0.5	0.4
Philippines	0.0	0.0	0.1	0.0	0.0	0.0	0.1	0.0
Somalia^a^	0.0	0.0	0.1	0.0	0.0	0.0	0.0	0.0
Sri Lanka	0.0	0.0	0.0	0.0	0.0	0.0	0.1	0.0
Tunisia	0.1	0.0	0.1	0.0	0.1	0.0	0.1	0.0
Vietnam	0.0	0.0	0.0	0.0	0.1	0.0	0.1	0.0
Zimbabwe	0.1	0.0	0.0	0.0	0.0	0.0	0.0	0.0
Total (%)^b^	**5.3**	**0.8**	**8.3**	**0.9**	**8.4**	**0.3**	**12.1**	**2.8**

^a^Somalia is the only low-income economy country in the table; all others are lower middle-income economy countries

^b^Percentage compared to the total submitted/published from all countries of the world for any given year

Although the numbers remain low, Table 1 suggests a trend towards increasing numbers of manuscripts being submitted to *Pediatric* *Radiology* from LMIC in 2022 compared to 2019, from 5.1% to 12.3%; note that this increase does not reach statistical significance (*P*=0.202) (IBM SPSS Statistics Version 29, IBM, Armonk, NY).

## Discussion

There is a low prevalence of first/last author positions from LMIC in published articles and in research based in LMIC or in research exploring global health, infectious disease and paediatrics. In addition, there is underrepresentation of radiologists from LMIC and LIC on the editorial boards of top-tier radiology journals and our own journal *Pediatric* *Radiology*. However, the situation is not straightforward. For example, authors of the review articles summarised above, by the very nature of their study designs and due to journal review and publication processes, could not and did not assess the number of manuscripts which were rejected and did not make it to publication—it is possible that there is a high rejection rate for articles with first/last authors from LMIC, similar to what we have observed for *Pediatric Radiology* (Table 1). *Pediatric* *Radiology* operates a double-blind review process, in which neither the reviewers nor the authors are aware of who each other are. Although some authors fail to fully anonymise their manuscripts, the number is too small to account for the increased rejection rate of manuscripts from LMIC and more likely explanations include the number and/or quality of the manuscripts (including language barriers) and/or the novelty of the research (for example in terms of scanner model for computed tomography or ultrasound and field strength of the magnetic resonance machine).

The lack of diversity amongst editorial board members is well-documented. For example, in 2019, of 144 editorial board members for the high-ranking neurology journals *The Lancet Neurology*, *Acta Neuropathologica*, *Nature Reviews Neurology* and *Brain* and *Annals of Neurology*, none was from a “developing country” [43]. A (slightly) less stark lack of diversity in editorial board membership has been demonstrated in international journals covering other fields such as psychiatry [44], spine [45] and anaesthetics [46]. It should be highlighted that direct comparisons between LIC/LMIC and UIC for editorial board and manuscript submission/publication rates do not reflect overall number of paediatric radiologists from the two groups. Clearly, the larger the pool of paediatric radiologists, the more representation there will be. Many LIC/LMIC do not have dedicated paediatric radiologists [47]. Nevertheless, despite fewer paediatric radiologists per general population in LIC/LMIC compared to UIC, we would expect representation of each of the world’s seven regions (as opposed to specific countries), both on editorial boards and for accepted manuscripts. Many radiologists from LIC/LMIC now receive subspecialist training in Europe, the USA and Canada—for example the partnership between Tikur Anbesssa Specialized Hospital, Ethiopia and the Children’s Hospital of Philadelphia [48]—and are board certified. Such individuals are suitably qualified to take on editorial board roles and should be encouraged to do so when positions become available.

A second confounder to consider is the medical “brain drain” due to authors from LMIC emigrating to HIC or returning to HIC of which they are citizens. For example, three of the authors of this manuscript are females of minority ethnic background who received their medical training in LMIC/UMIC and yet are now affiliated to institutions in HIC. Therefore, although it is one indicator, the “functional diversity” of an editorial board (by which we mean the way in which it thinks and operates and therefore potentially influences the diversity of manuscripts that are accepted) cannot solely be represented by the current country of affiliation of its members.

**Fig. 4:**
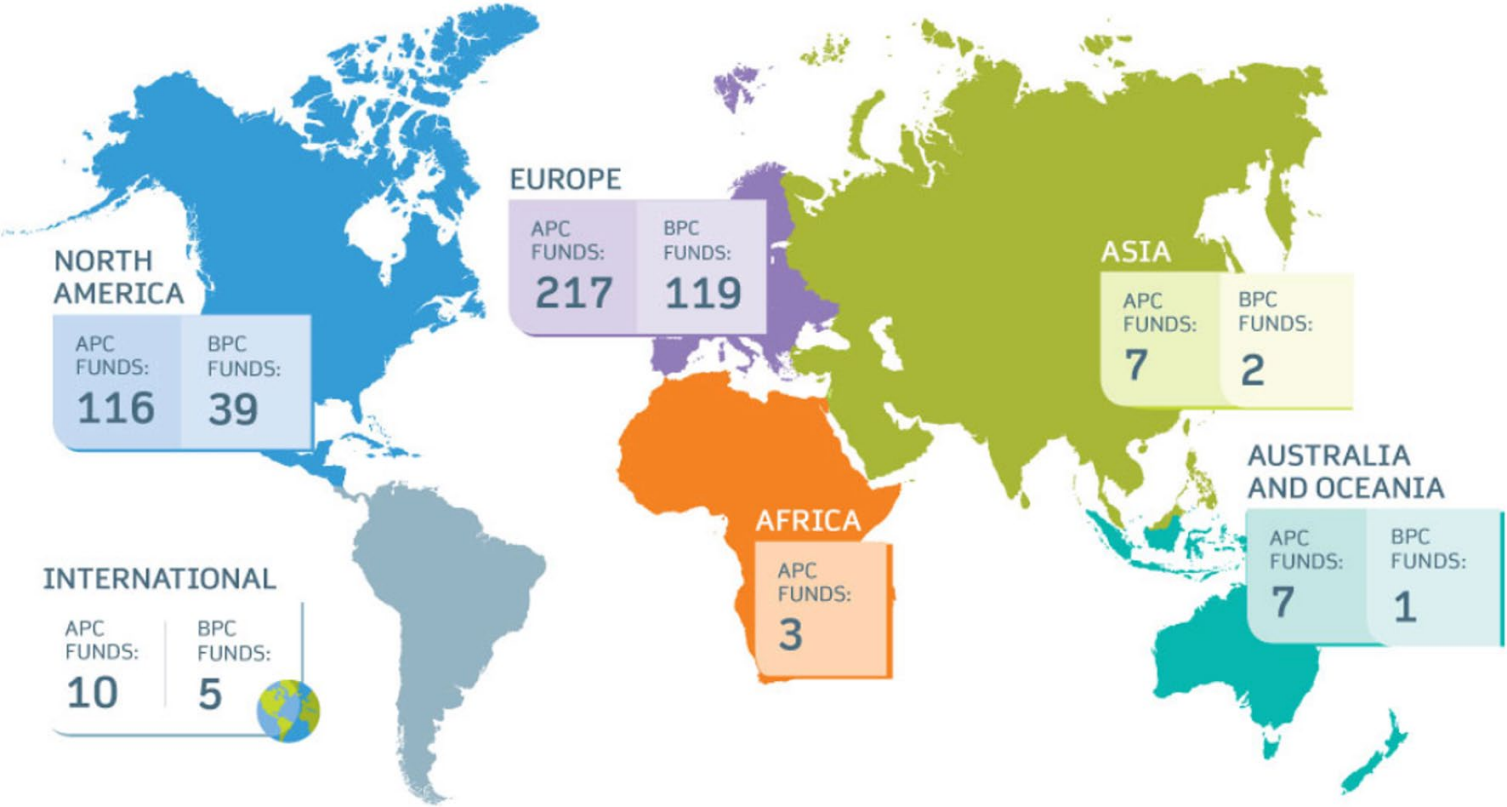
Springer Nature Open Access Map [50]. The map shows the number of funders/institutions that cover Springer Nature open access funds *APC* article processing charges, *BPC* book processing charges (published with permission from © Springer Nature. Licence Reference RP2023327)

Other issues to be considered include the cost of publications and access to publications. Access to published science is critical for advancing knowledge and for conducting future studies. Open access publication has been shown to be essential to LIC/LMIC, with authors of one paper calling for more sustainable open access dissemination channels so that there is less dependence on the private for-profit organisations, the goodwill of publishers and the willingness and/or ability of communities to pay [49]. Springer Nature provides open access to *Pediatric* *Radiology* through Transformative Agreements with institutions and funders under a Creative Commons Attribution 4.0 International licence. However, the number of countries in LIC/LMIC with this agreement in place is relatively small, as shown by the open access funds map available on the Springer Nature website (Fig. 4). Nevertheless, Springer’s open access journals waived fees of almost €20 million to authors in financial need in 2022, including almost €7 million for articles with corresponding authors based in LIC and LMIC [51].

Research4Life, an initiative with a mission to provide institutions in LIC/LMIC with equitable access to academic and professional peer-reviewed content, provides access to trustworthy and relevant peer-reviewed data free or at reduced cost to LIC/LMIC [52]. Currently, Research4Life has 1,584 partners, including societies (1,381) and publishers (172). No paediatric radiology society is a member. Springer Nature is one of the 172 publishers that are a Research4Life partner. Through its membership of Research4Life, Springer provides access to the Research4Life training portal, massive open online courses, webinars and other training resources. Through Research4Life, Springer Nature offers free online access to all its journals to researchers in eligible countries [53] and to Nature Masterclass online training content for researchers in LIC and LMIC [54].

Finally, there is limited evidence concerning the aspirations and requirements of researchers who are based in LMIC. It may be that authorship in high-ranking international journals is of sufficient status to meet local requirements for career success, regardless of author position, and that first/last authorship is of importance only to academics and academic institutions in HIC. Or it may be that manuscript authorship is not a criterion for academic success at all, and academic promotions are based purely on the number of years in service. Based on our experience, researchers from LIC/LMIC do value both first/last authorship positions and publication in high-impact journals. However, reasons for their failure/inability to hold first or senior author positions in journals of high impact are multiple—first, authors who have emigrated to HIC obtain the grants to conduct research in LIC/LMIC but take the senior author roles (it should be noted that eligibility for some grants is dependent on citizen status); second, authors from LIC/LMIC become disillusioned with the high rejection rate of their papers (for whatever reason) and stop submitting their manuscripts to (high impact) international journals; third, authors may be disincentivised by the high cost of publication. Finally, it is difficult in a technical specialty such as radiology to produce cutting-edge, internationally competitive research using older models of equipment, which may be the norm in LMIC and LIC, as such manuscripts may be less competitive than those from UMIC and therefore rejected.

In conclusion**,** although there is a lack of diversity throughout the research cycle related to gender, race/ethnicity, socioeconomic status, academic affiliation and other factors, this article has focused on diversity as it relates to national income. Over the past four years, 2019–2022 inclusive, only 8% of manuscripts submitted to and 1% of manuscripts published in *Pediatric* *Radiology* have come from LIC/LMIC (the frequency of acceptance of manuscripts from UMIC was seven times higher than that from LMIC (no manuscripts were published from LIC)). Only 2% of our editorial board members are from a LIC/LMIC and none of the societies we represent is currently a Research4Life partner. Increased collaboration is required between researchers across the globe to better understand the barriers to equity in the publication of research from LIC and LMIC and to identify ways in which we can overcome them together. With the ever-shrinking world, we believe that it is to everyone’s benefit to diversify research and publications and readers are directed to the accompanying editorial in this special issue [17] for initiatives that the current editors of *Pediatric* *Radiology* suggest should be put in place that might lead to the desired equity.

## Data Availability

Not applicable to this review of the literature.
